# Microsecond-sustained lasing from colloidal quantum dot solids

**DOI:** 10.1038/ncomms9694

**Published:** 2015-10-23

**Authors:** Michael M. Adachi, Fengjia Fan, Daniel P. Sellan, Sjoerd Hoogland, Oleksandr Voznyy, Arjan J. Houtepen, Kevin D. Parrish, Pongsakorn Kanjanaboos, Jonathan A. Malen, Edward H. Sargent

**Affiliations:** 1 Department of Electrical and Computer Engineering, University of Toronto, 10 King's College Road, Toronto, Ontario, Canada M5S 3G4; 2 Optoelectronic Materials Section, Department of Chemical Engineering, Delft University of Technology, Julianalaan 136, Delft 2628 BL, The Netherlands; 3 Department of Mechanical Engineering, Carnegie Mellon University, 5000 Forbes Avenue, Pittsburgh, 15213 Pennsylvania, USA; 4 Department of Material Science and Engineering, Carnegie Mellon University, 5000 Forbes Avenue, Pittsburgh, 15213 Pennsylvania, USA

## Abstract

Colloidal quantum dots have grown in interest as materials for light amplification and lasing in view of their bright photoluminescence, convenient solution processing and size-controlled spectral tunability. To date, lasing in colloidal quantum dot solids has been limited to the nanosecond temporal regime, curtailing their application in systems that require more sustained emission. Here we find that the chief cause of nanosecond-only operation has been thermal runaway: the combination of rapid heat injection from the pump source, poor heat removal and a highly temperature-dependent threshold. We show microsecond-sustained lasing, achieved by placing ultra-compact colloidal quantum dot films on a thermally conductive substrate, the combination of which minimizes heat accumulation. Specifically, we employ inorganic-halide-capped quantum dots that exhibit high modal gain (1,200 cm^−1^) and an ultralow amplified spontaneous emission threshold (average peak power of ∼50 kW cm^−2^) and rely on an optical structure that dissipates heat while offering minimal modal loss.

## Abstract (web summary)

To date, lasing in colloidal quantum dot solids has been limited to the nanosecond temporal range, limiting the potential for solution-processed lasers. Here, the authors combine thermal management with low amplified spontaneous emission threshold to produce microsecond-sustained lasing.

## Graphical abstract

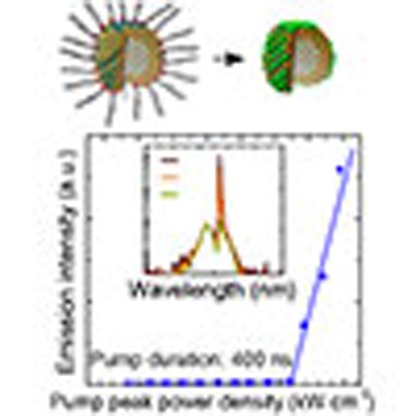

Colloidal quantum dots (CQDs) have attracted interest in light emission applications because of their high photoluminescence quantum yield^1^, size-controlled wavelength tunability across the visible^2^ and infrared spectral spectral regimes^3^, and convenient processing from the solution phase. They can provide amplified spontaneous emission (ASE) and lasing, but to date sustained only on the femtosecond^4^,^5^,^6^,^7^ to nanosecond timescales^8^,^9^,^10^. This prevents their application when more sustained population inversion is required.

Much prior work has begun from the assumption that Auger recombination is the most important effect that limits CQD lasing^11^. As a result, there has been an intense focus on extending the Auger lifetime using core–shell structures^12^. However, previous works have already seen lasing sustained on the nanosecond timescale, a duration already longer than reported Auger lifetimes^11^,^13^.

We sought to determine, and then remedy, what had previously curtailed sustained ASE and lasing. One possible mechanism is the effect of temperature beyond nanosecond times. Here a number of effects could work together. Much of the energy of the pump-produced photoexcitation is lost through thermalization and also non-radiative recombination—and all such losses contribute to heating of the film. In prior reports, both the CQD film and also the substrates employed have suffered from low thermal conductivity. Given that Auger recombination is highly thermally activated^14^, these two effects together could give way to a runaway increase in threshold and loss of ASE once the film temperature has risen due to continued heating over the duration of an extended excitation pulse. Keeping these considerations in mind, we develop ultra-compact CQD films to achieve record-low lasing threshold measured in peak power enabling microsecond-sustained lasing. We conclude with a discussion of the path to continuous-wave (CW) lasing and light amplification from CQD solids.

## Results

### Compact inorganic-halide-capped CQD films

**Figure 1:**
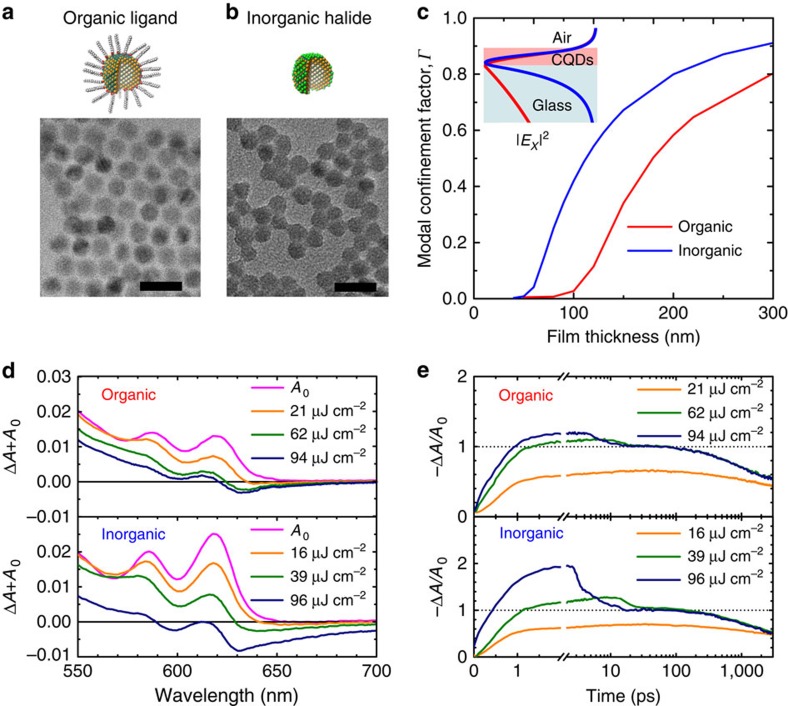
Modal confinement and material gain of inorganic-halide-capped CQDs. Illustration and transmission electron microscopy image of (**a**) organic-ligand- and (**b**) inorganic-halide-capped CQDs. Scale bar, 20 nm. The replacement of long-chained ligands with inorganic halides reduces interparticle distance leading to an increase in film refractive index. The average core–core distance (*d*_core–core_) between nanocrystals is 10.5 nm in organic-ligand-capped and 8.0 nm in inorganic-halide-capped CQDs. (**c**) The simulated dependence of the modal confinement factor, *Γ* (ratio of electric field |*E*_*x*_|^2^ in the CQD film) for organic-ligand- (red) and inorganic-halide (blue)-capped CQDs on glass substrate as a function of film thickness. The modal electrical field profile is calculated using refractive indices of *n*_organic_=1.74 and *n*_inorganic_=1.97 (Supplementary Fig. 1). The threshold condition is given by *g*_modal_*=Γ g*_material_*−α*_i_*=0*, where *g*_material_ is the material gain and *α*_i_ is the internal loss^20^. The shorter interparticle distance in inorganic-halide-capped CQDs therefore facilitates lower ASE threshold per film thickness. The inset shows the simulated normalized electric field profile in an organic- (red) and inorganic-halide (blue)-capped film with a thickness of 120 nm on a glass substrate. (**d**) The OD (Δ*A+A*_0_) as a function of pump fluence (180 fs, *λ*=400 nm) for organic-ligand- and inorganic-halide-capped CQDs on a glass substrate taken 3 ps after excitation. The optical gain threshold is 21 μJ cm^−2^ for organic-ligand-capped dots and 16 μJ cm^−2^ for inorganic-halide-capped dots. The ASE threshold, which must overcome internal losses is ∼62 and 39 μJ cm^−2^, for organic-ligand- and inorganic-halide-capped CQDs, respectively. (**e**) Pump-fluence-dependent transient absorption (−Δ*A/A*_0_) averaged over the 1S peak. A rapid change in absorption occurs between 3 and 25 ps caused by stimulated radiative recombination (that is, ASE). Increasing pump fluence further above threshold results in increased stimulated recombination between 4 ps and 25 ps. After the stimulated recombination process, the transient absorption reaches transparency, regardless of the fluence (above threshold). This can be observed by the overlapping transient absorption curves between 25 and 3,000 ps.

We prepared a population of compact CdSe–CdS–ZnS core–shell–shell dots (Fig. 1a) that leveraged a recently reported CdSe–CdS synthesis protocol having record-high photoluminescence quantum yield, narrow spectral linewidths and suppressed blinking^1^. In light of the possibility that heat dissipation was the most pressing issue in sustained CQD lasing, we began from the same quantum dots, but took measures to increase the chances of sustained lasing. We reasoned that, in light of the low thermal conductivity of even the best CQD solids^15^, producing the thinnest possible gain-providing film would benefit sustained gain and lasing. This would require maximal densification of the CQD film to maintain sufficient optical modal confinement.

Inorganic ligands have been employed in producing CdSe CQD films with a high electron mobility of *μ*_e_≈12 cm V^−1^ s^−1^ (ref. 16). However, their preservation of extended excited state lifetimes, have not been investigated. Further, they typically rely on the use of high-boiling-point solvents such as *n*-methyl-formamide^16^ and dimethyl sulfoxide^17^, which makes forming thick high-optical-quality (smooth and crack free) films very challenging. The alternative to solution exchange—layer-by-layer solid-state exchange—can often lead to cracking caused by the reduction in interparticle spacing during substrate-based ligand removal preparation^18^,^19^. Such morphological discontinuities scatter guided modes out of the film and increase modal loss.

We developed a new non-phase-transfer chloride ligand exchange using SOCl_2_ (Fig. 1b) that would overcome these limitations. The exchange from a long-chain organic surfactant to the chloride ligand was compatible with the use of low-boiling-point acetonitrile as film-forming solvent, enabling the formation of thick films with excellent thickness uniformity from a single spin cast.

The chloride ligand should result in a higher density of CQDs: 2.2 × 10^18^ cm^−3^ in the inorganic case compared with 1.0 × 10^18^ cm^−3^ in the organic-capped case in light of the smaller average core–core distance between dots (8.0 versus 10.5 nm, Methods).

We expected that this would produce a notably higher refracted index. Indeed, the inorganic film was measured to have refractive index of 1.97±0.05 compared with 1.74±0.05 for the organic film (all at emission wavelength *λ*=630 nm, see Supplementary Fig. 1). The same modal confinement factor, *Γ*, defined as the fraction of modal intensity |***E***_***x***_|^2^ in the CQD film 
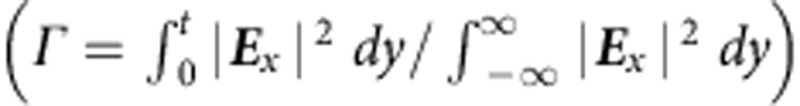
, is predicted to be realized in inorganic films that are at least 50% thinner than their organic liganded counterparts (Fig. 1c). The high modal confinement factor directly benefits the lasing threshold condition *g*_modal_=*Γ g*_material_−*α*_i_*=0*, where *g*_material_ is the material gain and *α*_i_ is the total modal loss^20^.

In femtosecond-pulsed studies, we found that the optical gain is indeed greater in inorganic-halide-capped CQDs than organic-ligand-capped CQDs (Fig. 1d) for films of similar thickness (120 and 135 nm, respectively). Δ*A+A*_0_ is the sum of change in absorption and the ground-state absorption, *A*_0_. The combination of greater modal confinement and higher material gain contribute to higher modal gain in much thinner inorganic-halide-capped films. In fact, the minimum thickness of CQD film on a glass substrate required to achieve ASE is 72±5 nm in the inorganic-halide-capped film compared with 120±10 nm in the organic-ligand-capped film (Supplementary Fig. 1).

The transient behaviour during stimulated emission and carrier decay can be observed from pump-fluence-dependent transient absorption averaged over the 1S peak (Fig. 1e). The rapid change in −Δ*A/A*_0_ occurs between 3 and 25 ps due to stimulated radiative recombination (that is, ASE). Increasing pump fluence further above threshold results in increased gain at short times, but after the stimulated recombination process (beyond 25 ps) the transient absorption reaches transparency (−Δ*A/A*_0_=1) regardless of the initial gain value. This can be seen via the overlapping transient absorption curves between 25 and 3,000 ps. One potential concern regarding the inorganic films is the possibility that the benefits to Auger lifetime could be degraded due to reduced passivation or increased interdot communication in these films. In actual fact, transient studies focused on the timescale between 25 and 1,000 ps after excitation (Supplementary Fig. 2) substantially overlap for the organic and inorganic films above threshold, confirming that the extended Auger lifetime of ∼700 ps was preserved in the inorganic processing case.

**Figure 2:**
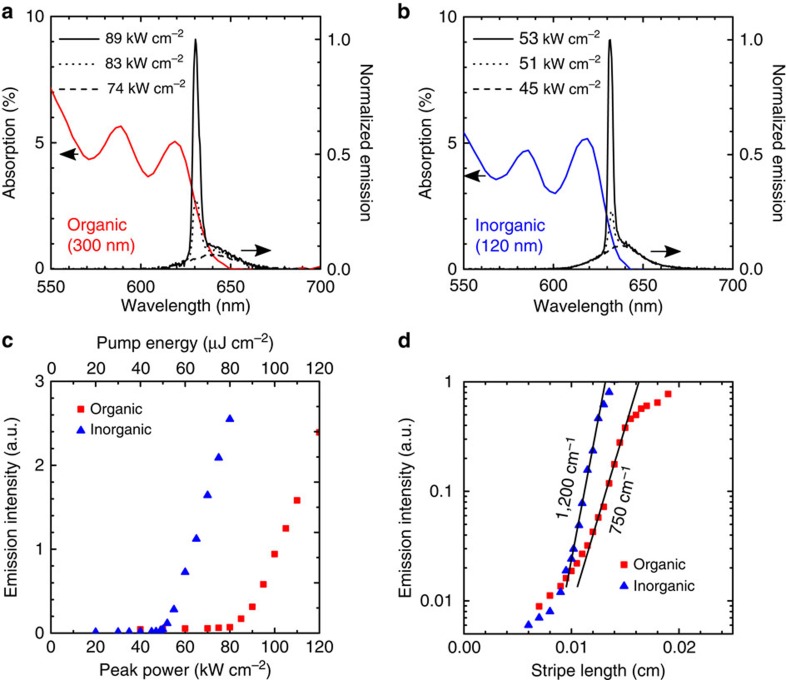
Optical gain in organic-ligand- and inorganic-halide-capped CQDs. Film absorption and edge emission spectra below and above ASE threshold for organic-ligand- (**a**) and inorganic-halide-capped (**b**) CQD films. The film thickness (300 nm for organic, 120 nm for inorganic) corresponds to approximately equal optical density at a pump wavelength of *λ*=355 nm. (**c**) Emission as a function of average pump peak power density or energy density. The ASE threshold is 83 kW cm^−2^ for organic-ligand-capped and 51 kW cm^−2^ for inorganic-halide-capped CQDs. (**d**) Modal gain measured by variable stripe length measurement for the organic-ligand- (750 cm^−1^) and inorganic-halide (1,200 cm^−1^)-capped CQD films excited at an average pump power density ^∼^4 × ASE threshold for each film.

We then moved to a nanosecond-duration transient photoexcitation to study ASE and its thresholds on a longer timescale. The main contributor here to carrier loss is Auger recombination, the same mechanism expected to dominate as we seek to approach CW operation (results of Monte Carlo simulations of the population contributions from single exciton, biexciton and multi-exciton population and loss contribution from recombination due to Auger, photoluminescence and traps are detailed in Supplementary Figs 4–6). Films were photoexcited using a stripe pump, and emission was collected from the edge of the sample (Methods). The thickness of inorganic and organic films was separately optimized to minimize ASE threshold in each case. For inorganic films, this corresponded to 120-nm-thick films that had an ASE threshold (Fig. 2a,b) of 51 kW cm^−2^ average peak power (51 μJ cm^−2^ per pulse), while for organic films as the optimum was 300 nm thickness, which produced ASE threshold 83 kW cm^−2^ average peak power (83 μJ cm^−2^ per pulse). The ASE thresholds, reported in average peak power, are the lowest reported in CQD solids^8^. The emission spectra are collected from the edge of the sample since ASE propagates laterally within the CQD film. Therefore, the PL portion of the emission spectra (small peak located at ∼640 nm) is attenuated by absorption in the film. The full PL spectra collected from the surface of the sample (Supplementary Fig. 7) show that the ASE peak is in fact redshifted with respect to the PL peak.

**Figure 3:**
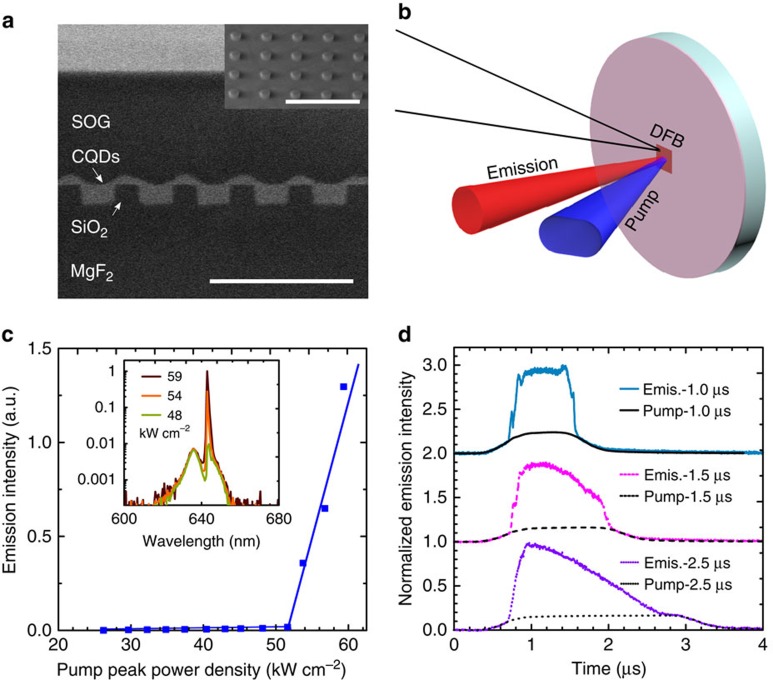
Microsecond-sustained lasing. (**a**) Cross section SEM image of a 2D distributed feedback (DFB) array structure on thermally conductive MgF_2_ substrate. The inset shows a 45° tilt SEM of the 2D array of cylinders before CQD film deposition. Scale bar, 1 μm. (**b**) Illustration of the measurement set-up. (**c**) Emission intensity as a function of the average excitation peak power showing a lasing threshold at 52 kW cm^−2^ and pump duration of 400 ns. The lasing spectra are shown in the inset. (**d**) Transient behaviour of the emission from the DFB structure for three different pump durations (1.0, 1.5 and 2.5 μs) at a pump power density of 88 kW cm^−2^. Lasing is sustained up to 1.8 μs. The intensity from the pump pulse is shown for comparison.

The inorganic film benefited from three separate advantages. The combination of (1) high material gain, (2) high modal confinement (Fig. 1c) and (3) minimal scattering due to low roughness and improved thickness uniformity (Supplementary Figs 8 and 9), together enabled the inorganic film to reach net modal gain at the record-low excitation intensity. Variable stripe length method^21^ studies of net modal gain (Fig. 2d) confirmed that under pump fluence ∼4 × ASE threshold for each film the net modal gain was 1,200 cm^−1^ for the (notably thinner) inorganic film, compared with 750 cm^−1^ for the organic.

### Microsecond-sustained lasing

Lower ASE threshold, minimal scattering and high refractive index are all advantageous properties to achieve lasing. We sought to apply the new CQD films to achieve sustained lasing. We used MgF_2_ as a transparent substrate in view of its high thermal conductivity compared with other transparent-in-the-visible materials (21 Wm^−1^ K^−1^ along the *c* axis and 30 Wm^−1^ K^−1^along the *a* axis^22^) and low refractive index (*n*=1.38) for supporting modal confinement in the CQD film. A two-dimensional (2D) distributed feedback (DFB) array of cylinders was designed and fabricated using electron-beam lithography (Fig. 3a). A 2D array was chosen over a one-dimensional (1D) array to maximize thermal contact between the CQD film and thermally conductive substrate. The resonant lasing wavelength is given by the Bragg expression: *λ*_bragg_=2*n*_eff_*Λ*/m, where *n*_eff_ is the effective index, *Λ* is the periodicity of the 2D array of cylinders and *m* is the order. The array is chosen to be a second-order DFB to produce emission directed normal to the surface of the substrate (Fig. 3b; Methods.). The spin-on-glass layer was added as a protective layer and it did not lower ASE threshold of the device (Supplementary Figs 10–12). The lasing threshold, measured using a pump pulse duration of 400 ns (Fig. 3c), occurs at 52 kW cm^−2^ average peak power density. The well-defined laser emission spectrum centred at *λ*=643 nm has full width at half maximum of 0.7 nm (Fig. 3c, inset). Lasing is sustained greater than 1 μs when excited at pump power density of 88 kW cm^−2^ (Fig. 3d). The duration of lasing reported herein is three orders of magnitude longer than that in previously reported CQD devices^8^.

### Thermal measurements and simulations

The new inorganic film, when appropriately integrated onto a suitably thermally conductive substrate, allowed the first realization of microsecond CQD lasing. This result motivated us to elaborate further the thermal model of the system. We would seek to quantify the relative roles of lowered heating due to lowered ASE threshold from the use of a smooth dense film, lowered heating due to a shorter dissipation distance in thin compact inorganic films, and higher thermal transport through the MgF_2_ substrate when compared with prior ns-only reports.

To enable the construction of a thermal model, we measured the thermal conductivity of each CQD film. We used the pump-probe optical technique frequency domain thermoreflectance (see Methods and Supplementary Figs 13 and 14 for description and details). The inorganic-halide-capped film had similar thermal conductivity (0.24±0.04 W m^−1^ K^−1^) to that of the organic-ligand-capped film (0.22±0.04 W m^−1^ K^−1^). These thermal conductivities are comparable with values reported previously in CdSe core-only quantum dots^15^.

**Figure 4:**
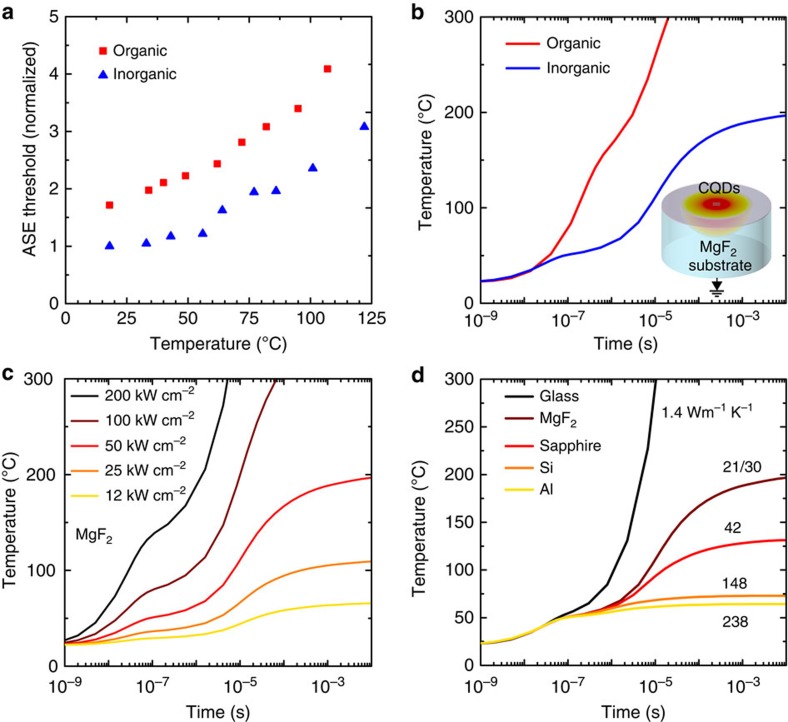
Effect of temperature on ASE threshold and temperature management. (**a**) Measured ASE threshold as a function of temperature, normalized to the ASE threshold of inorganic CQDs at room temperature. (**b**) Simulated film temperature as a function of time for an organic-ligand-capped film (ASE threshold=83 kW cm^−2^, thickness=300 nm) and inorganic-halide-capped film (ASE threshold=51 kW cm^−2^, thickness=120 nm) on a thermally conductive MgF_2_ substrate. The inset illustrates the heat dissipation profile in the CQD film. (**c**) Simulated film temperature as a function of time for different ASE thresholds. The substrate is MgF_2_ for each threshold. (**d**) Simulated film temperature as a function of time for different substrates. The pump power is 50 kW cm^−2^ for each substrate. Decreasing the ASE threshold and choice of substrate are critical factors in achieving microsecond lasing. A path towards continuous-wave lasing would involve further decreasing the ASE threshold/changing to a substrate such as Si.

Our modelling study also required knowledge of the temperature dependence of the ASE threshold. We obtained this in the athermal (fs) regime and with the aid of heatsink-based control of substrate temperature (Fig. 4a). The ASE threshold of the two films is normalized to the room temperature ASE of the inorganic-halide-capped film. The two materials had similar relative thermal dependencies of threshold, as expected since, in the athermal regime, the lifetime associated with Auger effect in non-degenerate and intrinsic semiconductors are dependent on temperature in the following way:


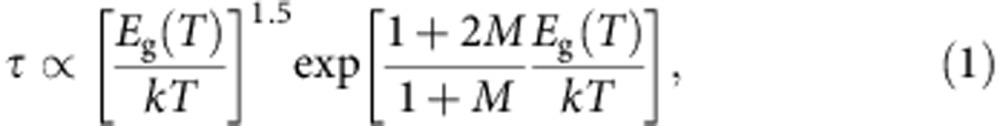


where *E*_g_ is the bandgap, *T* is the temperature and *M* is the electron–hole mass ratio^23^.

We then deployed the model with the goal of accounting for a number of key experimental observations. First, we explore the failure to achieve microsecond lasing of the organic film. The simulated temperature as a function of time shows a marked difference for the inorganic- and organic-capped films both on MgF_2_ substrate (Fig. 4b). We estimate that the device temperature of the inorganic CQD laser presented in Fig. 3 is ∼60 °C after 2 μs (Fig. 4b) at which the ASE threshold is ∼1.7 × above room temperature conditions (Fig. 4a). The difference in temperature can be explained by two of the above-mentioned factors: lower threshold and shorter heat dissipation distance in the inorganic-halide-capped film. The ASE threshold has the greatest impact on the film temperature (Fig. 4c). A thinner film (assuming equal optical density at the pump wavelength for each film thickness) also assists in lowering the film temperature in light of the low thermal conductivity of the CQD films (Supplementary Fig. 15). After instantaneous top illumination of a quantum dot film using pump light, unconverted energy generates heat, and we estimate that this diffuses through the film and reaches the substrate after *τ* ∼70 ns (thermal analysis detailed in Supplementary Note 1: Thermal Analysis). We proceeded to simulate the effect of substrate thermal conductivity on the temperature of the CQDs (Fig. 4d). The lower heating due to lower threshold, shorter heat dissipation distance and also the judicious choice of substrate for thermal transport were all quantitatively appreciable contributing factors for achieving the first report of microsecond lasing. When each of the parameters (1) ASE threshold, (2) film thickness and (3) substrate thermal conductivity is independently changed by ∼2 × from the baseline case (50 kW cm^−2^ ASE threshold, 120 nm thickness, MgF_2_ substrate), the resulting changes in steady-state temperature are (1) 80%, (2) 14% and (3) 50%, respectively.

## Discussion

These findings suggest a pathway to CW lasing using CQD solids. Further decreasing the ASE threshold, combined with employing, a more thermally conductive substrates such as Al, should, if implemented without compromise to optical properties, enable CW lasing. Substrates such as Al and Si absorb visible light, so a suitably thin, thermally conductive, low-index cladding between the substrate and the active layer will be required.

The development of ultra-compact thin films herein was the crucial enabler of microsecond-sustained lasing. Thermal simulations suggest that further progress towards CW lasing from CQD solids will benefit from a further decrease in ASE threshold and from moving to a highly thermally conductive substrate that also supports low-loss optical propagation. The inorganic halide exchange developed herein provides the added benefit that it is performed in solution, allowing films to be deposited directly without additional post-process washing or solid-state ligand exchange steps. The inorganic-halide-exchanged, directly deposited CQD films offer additional advantages relative to organically capped CQDs, since related reports suggest higher electron mobility for electrical injection^16^, and the removal of organics is expected to produce higher structural rigidity as well as the observed increased film thickness uniformity. These advantages will be helpful for the development of optoelectronic applications such as LEDs and electrically injected lasers.

## Methods

### Chemicals

Cadmium oxide (CdO, >99.99%), zinc acetate dihydrate (Zn(AC)_2_· 2H_2_O, 99.99%), sulfur powder (S, >99.5%), selenium powder (Se, >99.99%), oleylamine (OLA, 80–90%), octadecene (ODE, 90%), oleic acid (OA, 90%), tri-octylphosphine (TOP, 90%), tri-butyl phosphine (TBP, 97%), tri-octylphosphine oxide (TOPO), octadecylphosphonic acid (ODPA, 97%), 1-octanethiol (>98.5%) and thionyl chloride (SOCl_2_) were purchased from Sigma Aldrich without further purification.

### CdSe CQD synthesis

CdSe CQDs were synthesized using existing literature protocol^24^. A amount of 240 mg CdO, 24 g TOPO and 1.12 g ODPA were mixed in a 100-ml three-neck flask, the mixture was heated to 150 °C for 0.5 h under vacuum, and the temperature was then brought to 320 °C and kept at that temperature for 2 h under nitrogen. A measure of 4 ml of TOP was injected into the flask and the temperature was further raised to 380 °C. The injected selenium precursor consisted of 2 ml selenium in TOP solution at a concentration of 60 mg ml^−1^. CQDs that exhibited an excitonic peak at 580 nm were produced as a result of ∼3-min growth. The reaction was terminated by removing the heating mantle and, adding acetone. The resultant nanoparticles were redispersed in hexane for shell growth.

### Cd-oleate and Zn-OLA complex

A amount of 2.98 g CdO was fully dissolved in 40 ml oleic acid at 170 °C under vacuum. A amount of 2.45 g Zn(AC)_2_·2H_2_O was dissolved in OLA at 170 °C under vacuum until a clear light pink solution was obtained.

### CdS and ZnS shell growth

The shell growth procedure here was developed from a recent publication^1^. CdSe CQDs were quantified by measuring the absorbance at peak exciton (580 nm) with 1-mm light-path length cuvette. A 8.8-ml core-only CQD dispersion with an optical density of 2 at the exciton peak was added into a mixture of 12 ml OLA and 12 ml ODE in a flask, and pumped in vacuum at 100 °C to remove hexane. A measure of 3 ml as-prepared Cd-oleate was diluted in 21 ml ODE and 320 μl octanethiol was diluted in 24 ml ODE. Cd-oleate and octanethiol solutions were injected simultaneously and continuously at a rate of 12 ml h^−1^ during the ramping of temperature from 100 to 310 °C.

After CdS shell growth, the solution was cooled down to 290 °C and held at this temperature for 10 min. A measure of 1.5-ml as-prepared Zn-OLA diluted in 10.5 ml ODE and 0.03 g sulfur dissolved in 2 ml OLA was mixed and continuously injected at a speed of 14 ml h^−1^ at 290 °C to grow the ZnS shell. The solution was annealed for 10 min at 290 °C, followed by an injection of 4 ml OA and a further anneal at 290 °C for 10 min.

CdSe–CdS–ZnS (core–shell–shell) CQDs were purified by three cycles of centrifugation at 6,000 r.p.m., precipitation by adding a mixture of acetone and method (volume ratio of 2:1), and redispersionin hexane. The final core–shell–shell CQDs were dispersed in toluene such that the peak exciton optical density was 2.5 (1-mm optical path length) for film fabrication.

### Chloride ligand exchange

A measure of 500 μl of the above CQDs in toluene solution was mixed with 1.25 ml TBP, followed by 500 μl SOCl_2_ in toluene solution (volume ratio of 20 μl SOCl_2_ ml^−1^ toluene). The CQDs precipitated within several minutes and the resulting solution was kept in the glovebox overnight to ensure complete exchange. TBP was necessary for the exchange to prevent CQD etching^25^.

After exchange, anhydrous hexane was added to completely precipitate the CQDs before centrifugation at 6,000 r.p.m. CQDs were washed with three cycles of adding anhydrous acetone to disperse the CQDs and adding hexane to precipitate the CQDs dispersion. The chloride ligands passivated CQDs were finally dispersed in 500 μl acetonitrile solution for film fabrication.

### Film preparation and laser fabrication

For ASE measurements, CQD films were deposited using a single spin coat at a spin speed of 300–1,000 r.p.m. for 60 s onto glass substrates. Inorganic-halide-capped films were exposed to air for 1 day before ASE characterization.

The distributed feedback 2D array was fabricated by first depositing a 130-nm-thick SiO_2_ film by magnetron sputtering onto a single-crystal MgF_2_ substrate. A thin layer of Poly(methyl methacrylate) (PMMA) (950 K A3) was spin coated at 5,000 r.p.m. for 60 s onto the substrate and cured at 180 °C for 60 s. The PMMA was coated with a thin layer (∼8 nm) of thermally evaporated aluminum for laser height alignment. The PMMA was patterned using a Vistec EBPG 5000+ E-beam lithography system into a 2D array of circles with a diameter of 160 nm and periodicity of 430 nm in both the horizontal and vertical directions. Twenty nanometre of Al was thermally evaporated to act as an etch mask. The substrate was soaked in acetone overnight to remove Al by lift-off process. The SiO_2_ was etched by reactive-ion-etch with CHF_3_ (20 s.c.c.m.) and O_2_ (5 sccm) gas at a chamber pressure of 30 mtorr and power of 150 W in a Trion Phantom etcher. The Al was removed using chromium etchant solution. Inorganic-halide-capped CQDs were spin coated onto the DFB array at a spin speed of 1,000 r.p.m. for 60 s. A thick layer of spin-on-glass (Filmtronics 500F), used for protection from condensation, was spin coated at 1,500 r.p.m. for 12 s and annealed in N_2_ at 100 °C for 10 min. The spin-on glass did not lower the minimum ASE threshold (Supplementary Figs 10–12).

### Laser characterization

The laser emission was measured as illustrated in Fig. 4b. Two 442-nm 3-W laser diodes, combined using mirrors, a quarter waveplate and a polarizing beamsplitter cube, were used as the pump. The CW pump was modulated using an acousto-optic-modulator (IntraAction Corp., rise time ∼300 ns). The output from the acousto-optic-modulator was synchronized with an optical chopper operating at a frequency of 30 Hz to reduce background signal. The pump beam was focused onto the sample to spot size of 30 × 50 μm. The emission was collected through two lenses into a single-mode fibre. The spectrum was measured using an Ocean Optics USB2000+ spectrometer. Transient measurements were taken by collecting the laser emission directly into a 1.8-mm diameter fibre coupled with a Si photodetector (Thorlabs DET 36A, rise time=14 ns). The photodetector response was measured using a 1-GHz oscilloscope, which was continuously acquiring data averaged over 12 pulses.

### ASE and variable stripe length measurements

ASE was measured using a 1-ns pulse duration laser with a wavelength of 355 nm and frequency of 100 Hz. A 20-cm focal length cylindrical lens was used to focus the beam to a stripe with dimensions of 1,000 × 10 μm. The sample was excited perpendicular to the surface of the film and the emission was collected parallel to the film surface from the edge of the sample. The emission was collected using two lenses into a 50 μm diameter multi-mode fibre. The emission spectrum was measured using an Ocean Optics USB2000+ spectrometer. The modal gain was measured using the variable stripe length method. The stripe width was 10 μm and the length was varied between 0 and 1,000 μm. The modal gain was determined by the ASE emission intensity versus stripe length relation using the equation *I(L)=A*[*e*^*gL*^−1]/*g*, where *I* is the ASE emission intensity, *A* is a constant proportional to spontaneous emission intensity, *g* is the modal gain and *L* is the stripe length^21^.

### Transient absorption measurements

A Light Conversion Pharos laser with an optical parametric amplifier (Orpheus, Lightconversion) and a Helios white-light transient absorption spectrometer from Ultrafast systems were used for pump-probe measurements. The pump beam was a 400-nm laser source with 180-fs pulse duration and 2.5 kHz repetition rate. Transient absorption spectra in the visible (450–900 nm) were recorded at a repetition rate of 5,000 Hz using broadband probe pulses from a sapphire crystal pumped by the 1,030 nm fundamental of the laser. To investigate the absorption difference between excited and non-excited samples, every second pump pulse was blocked with a mechanical chopper resulting in a 2.5 kHz pump rate and a 5 kHz probe rate. To correct for chirp on the probe pulse, a bare glass substrate was measured. The time-zero was determined for each wavelength based on the coherent artifact. The time trace is taken by integrating the Δ*A* over the 1S peak.

### Thermal conductivity measurements

The thermal conductivity of the organic-ligand-capped and inorganic-halide-capped dots was measured using frequency domain thermoreflectence, a non-destructive CW laser technique. The samples consisted of multilayer thin films: a 100-nm gold transducer sputtered onto either an organic-ligand-capped quantum dot film or an inorganic-halide quantum dot film ranging from 128 to 242 nm in thickness on a silicon substrate. A 488-nm ‘pump' laser is intensity modulated and used to periodically heat the surface (gold layer) of the sample. The periodic temperature change, and hence reflectance change, induced by the pump causes a colinear 532-nm ‘probe' laser to reflect off the surface with a periodic intensity that has an amplitude and phase relative to the pump, which depend on the thermal transport properties of the underlying sample. Both the pump and probe signals are measured using a radiofrequency lock-in amplifier. The pump laser is modulated at different heating frequencies, resulting in different phase shifts between the pump and probe frequency responses. By fitting the experimental phase shift versus heating frequency to that predicted with an exact analytical solution to the heat diffusion equation^26^,^27^, we can extract the thermal conductivity of the quantum dot layer. Raw data and sample fits can be found in Supplementary Tables 1 and 2.

### Thermal simulations

ANSYS transient thermal software package was used to simulate the transient thermal response of our light-emitting systems during operation. Similar to our experiments, we study CQD thin films on bulk substrates and use a pump-beam spot size of 50 × 30 μm. Uniform thermal energy generation is considered throughout the volume of the CQD film within the pump spot area. This assumption is reasonable for the low optical density films studied in this work. The substrate backside is maintained throughout the simulation at the system initial temperature of 295 K. We choose bulk-like substrate thicknesses such that the propagating thermal wave decays to *T*=295 K well before it reaches the substrate backside. We allow the system to evolve in time and report the maximum temperature of the CQD film. Thermal simulation input parameters are reported in Supplementary Table 2.

### Absorption, photoluminescence and transient PL measurements

Photoluminescence and transient photoluminescence measurements were carried out on a Horiba FlouroLog-3 spectrofluorometer. The sample was excited with a 375-nm pulsed laser diode (<1 ns) and the emission passed through a 500-nm blaze grating monochromator and collected by a visible photomultiplier tube. All absorption measurements were collected using a Perkin Elmer Lambda 950 UV-Vis-NIR spectrophotometer equipped with an integrating sphere. Samples were place at the centre of the integrated sphere tilted at a tilt angle of 20° relative to the incident beam. The total transmission (*T*) and reflectance (*R*) were collected by the integrating sphere detector and the absorption was calculated as 100%−*T*−*R*. The 100% transmission baseline measurement was an empty sphere.

### Transmission electron microscopy measurements

Transmission electron microscopy images were taken by JEOL 2010, with an acceleration voltage of 200 kV. The CQD solution was diluted in toluene or acetonitrile and dropped onto ultrathin carbon support film on copper transmission electron microscopy grids. The density of CQDs is calculated based on the average core–core distance and assuming a packing fraction of 0.6 (where close packing is 0.74).

## Additional information

**How to cite this article:** Adachi, M. M. *et al*. Microsecond-sustained lasing from colloidal quantum dot solids. *Nat. Commun.* 6:8694 doi: 10.1038/ncomms9694 (2015).

## Supplementary Material

Supplementary Information Supplementary Figures 1-15, Supplementary Tables 1-2, Supplementary Note 1, Supplementary Methods and Supplementary References.

## References

[b1] Chen O. . Compact high-quality CdSe-CdS core-shell nanocrystals with narrow emission linewidths and suppressed blinking. Nat. Mater. 12, 445–451 (2013). 23377294 10.1038/nmat3539 PMC3677691

[b2] Murray C. B., Norris D. J. & Bawendi M. G. Synthesis and characterization of nearly monodisperse CdE (E=sulfur, selenium, tellurium) semiconductor nanocrystallites. J. Am. Chem. Soc. 115, 8706–8715 (1993).

[b3] Hines M. A. & Scholes G. D. Colloidal PbS nanocrystals with size-tunable near-infrared emission: observation of post-synthesis self-narrowing of the particle size distribution. Adv. Mater. 15, 1844–1849 (2003).

[b4] Dang C. . Red, green and blue lasing enabled by single-exciton gain in colloidal quantum dot films. Nat. Nano 7, 335–339 (2012). 10.1038/nnano.2012.61 22543426

[b5] Klimov V. I. & Bawendi M. G. Ultrafast carrier dynamics, optical amplification, and lasing in nanocrystal quantum dots. MRS Bull. 26, 998–1004 (2001).

[b6] Dang C. . Highly efficient, spatially coherent distributed feedback lasers from dense colloidal quantum dot films. Appl. Phys. Lett. 103, 171104 (2013).

[b7] Guzelturk B. . Stable and low-threshold optical gain in CdSe/CdS quantum dots: an all-colloidal frequency up-converted laser. Adv. Mater. 27, 2741–2746 (2015). 25807924 10.1002/adma.201500418

[b8] Guilhabert B. . Nanosecond colloidal quantum dot lasers for sensing. Opt. Express 22, 7308–7319 (2014). 24664078 10.1364/OE.22.007308

[b9] Schäfer J. . Quantum dot microdrop laser. Nano Lett. 8, 1709–1712 (2008). 18471023 10.1021/nl080661a

[b10] Chen Y. . Colloidal quantum dot random laser. Opt. Express 19, 2996–3003 (2011). 21369124 10.1364/OE.19.002996

[b11] Klimov V. I., Mikhailovsky A. A., McBranch D. W., Leatherdale C. A. & Bawendi M. G. Quantization of multiparticle auger rates in semiconductor quantum Dots. Science 287, 1011–1013 (2000). 10669406 10.1126/science.287.5455.1011

[b12] García-Santamaría F. . Suppressed Auger recombination in ‘giant' nanocrystals boosts optical gain performance. Nano Lett. 9, 3482–3488 (2009). 19505082 10.1021/nl901681d PMC2897714

[b13] Fisher B., Caruge J. M., Zehnder D. & Bawendi M. Room-temperature ordered photon emission from multiexciton states in single CdSe core-shell nanocrystals. Phys. Rev. Lett. 94, 087403 (2005). 15783930 10.1103/PhysRevLett.94.087403

[b14] Javaux C. . Thermal activation of non-radiative Auger recombination in charged colloidal nanocrystals. Nat. Nano 8, 206–212 (2013). 10.1038/nnano.2012.260 23396313

[b15] Ong W.-L., Rupich S. M., Talapin D. V., McGaughey A. J. H. & Malen J. A. Surface chemistry mediates thermal transport in three-dimensional nanocrystal arrays. Nat. Mater 12, 410–415 (2013). 23503009 10.1038/nmat3596

[b16] Zhang H., Jang J., Liu W. & Talapin D. V. Colloidal Nanocrystals with Inorganic Halide, Pseudohalide, and Halometallate Ligands. ACS Nano 8, 7359–7369 (2014). 24988140 10.1021/nn502470v

[b17] Kovalenko M. V., Scheele M. & Talapin D. V. Colloidal nanocrystals with molecular metal chalcogenide surface ligands. Science 324, 1417–1420 (2009). 19520953 10.1126/science.1170524

[b18] Zhang H. . Surfactant ligand removal and rational fabrication of inorganically connected quantum dots. Nano Lett. 11, 5356–5361 (2011). 22011091 10.1021/nl202892p

[b19] Luther J. M. . Structural, optical, and electrical properties of self-assembled films of PbSe nanocrystals treated with 1,2-ethanedithiol. ACS Nano 2, 271–280 (2008). 19206627 10.1021/nn7003348

[b20] Coldren L. A., Corzine S. W. & Mašanović M. L. in Diode Lasers and Photonic Integrated Circuits 2nd edn John Wiley & Sons, Inc. (2012).

[b21] Shaklee K. L., Nahory R. E. & Leheny R. F. Optical gain in semiconductors. J. Lumin. 7, 284–309 (1973).

[b22] Tropf W., Thomas M. F. & Harris T. J. Properties of Crystals and Glasses McGraw-Hill (1995).

[b23] Beattie A. R. & Landsberg P. T. Auger effect in semiconductors. Proc. R. Soc. Lond. A Math. Phys. Eng. Sci. 249, 16–29 (1959).

[b24] Yu W. W., Qu L., Guo W. & Peng X. Experimental determination of the extinction coefficient of CdTe, CdSe, and CdS nanocrystals. Chem. Mater. 15, 2854–2860 (2003).

[b25] Anderson N. C. & Owen J. S. Soluble, chloride-terminated CdSe nanocrystals: ligand exchange monitored by 1H and 31P NMR spectroscopy. Chem. Mater. 25, 69–76 (2013).

[b26] Cahill D. G. Analysis of heat flow in layered structures for time-domain thermoreflectance. Rev. Sci.c Instrum. 75, 5119–5122 (2004).

[b27] Malen J. A. . Optical measurement of thermal conductivity using fiber aligned frequency domain thermoreflectance. J. Heat Transfer 133, 081601–081601 (2011).

