# Supplementary material for: Microsecond-sustained lasing from colloidal quantum dot solids
Source: Nat Commun. 2015 Oct 23;6:8694. doi: 10.1038/ncomms9694 (PMC4846322; doi:10.1038/ncomms9694)
Supplement: Supplementary Information — Supplementary Figures 1-15, Supplementary Tables 1-2, Supplementary Note 1, Supplementary Methods and Supplementary References. [file ncomms9694-s1.pdf]

Supplementary Figures

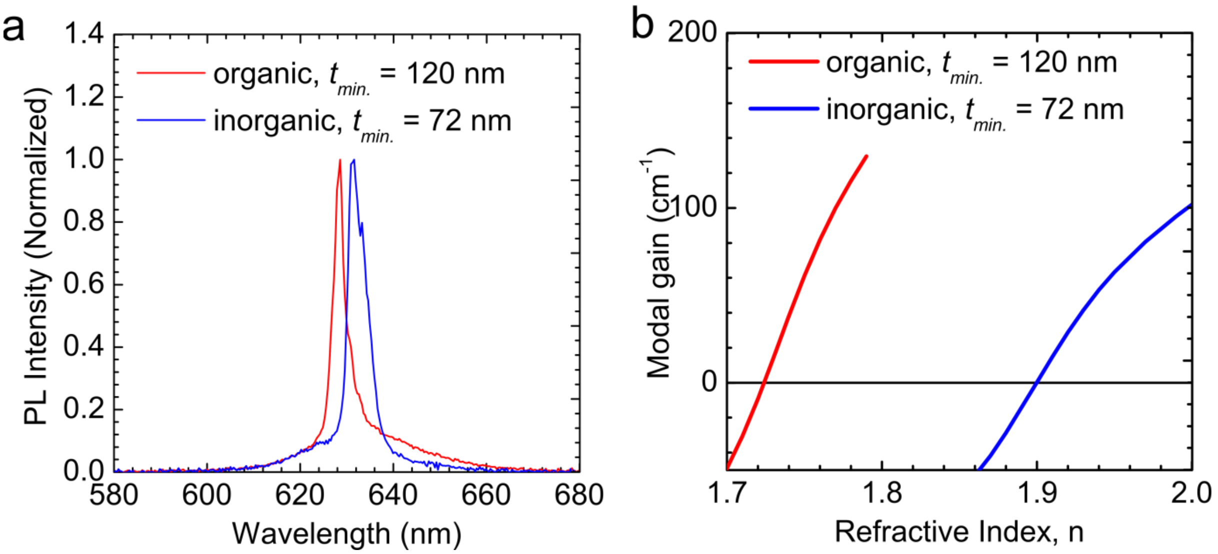

Supplementary Figure 1: Refractive index based on the minimum film thickness (t_{min}) to achieve ASE. (a) The ASE spectra measure for a $t_{min.}$= 120 nm for the organic-ligand capped CQDs and $t_{min.}$= 72 nm for the inorganic-halide capped CQDs. (**b**) The modal gain calculated as $g_{modal} = \Gamma g_{material} - \alpha_i$, where Γ is the confinement factor, $g_{material}$ is the material gain and α_i is the internal loss. Γ is simulated by a 1D wave solver (Lumerical Mode Solutions) and α_i is the simulated propagation loss. $g_{material}$ is taken from Fig. 1c, right above ASE threshold for each material.

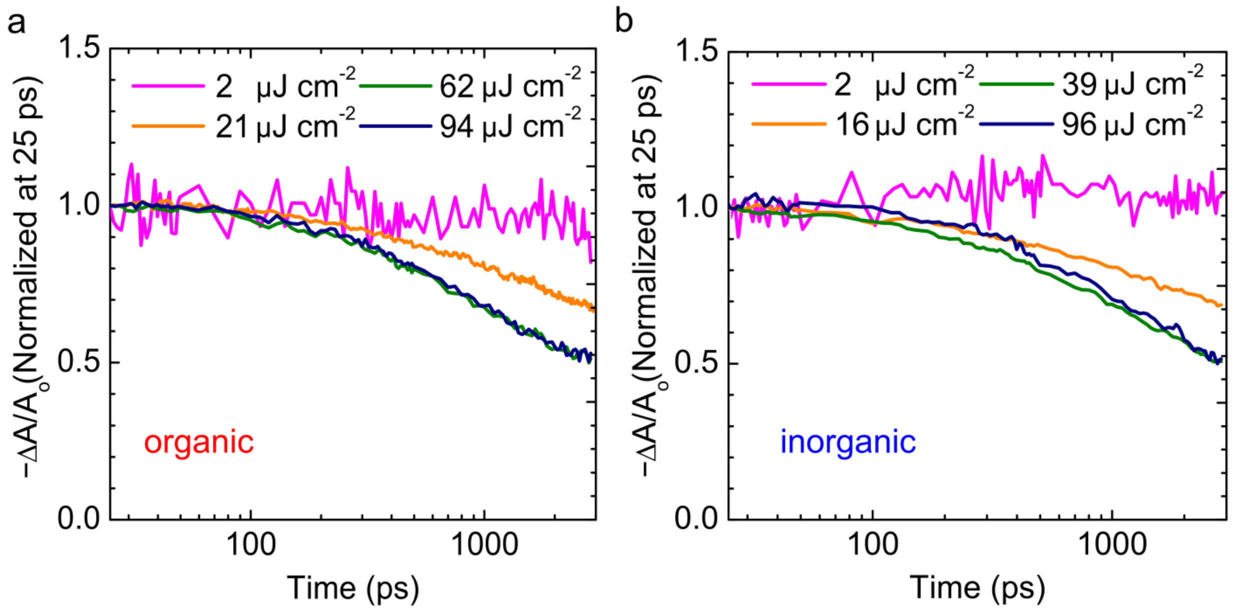

Supplementary Figure 2: Pump fluence dependent transient absorption normalized to 25 ps after excitation (180 fs excitation pulse). The decay rate increases for increasing pump fluence until ASE is reached, after which the decay curves after the ASE process (after 25 ps) overlap. Above ASE threshold the decay profile of the organic (62 μJ cm^{-2}) and inorganic (39 μJ cm^{-2}) films are almost identical confirming that the Auger lifetime (~700 ps, calculated to be the fast component of a bi-exponential fit) was preserved in the inorganic processing case.

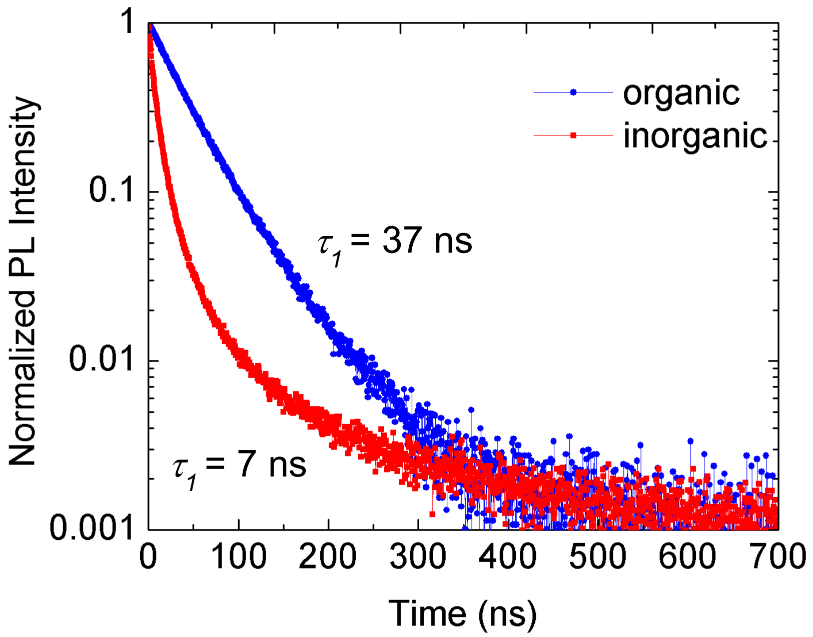

Supplementary Figure 3: Photoluminescence transient response of the inorganic-halide and organic-ligand capped films. The removal of long chained ligands leads to more efficient dissociation in the inorganic-halide capped CQDs. As a result the dominant PL time constant, taken as the time at which the normalized PL intensity decreases to 1/e, is significantly shorter in the inorganic-halide capped dots (τ_1 = 7 ns) than the organic-ligand capped dots (τ_1 = 37 ns).

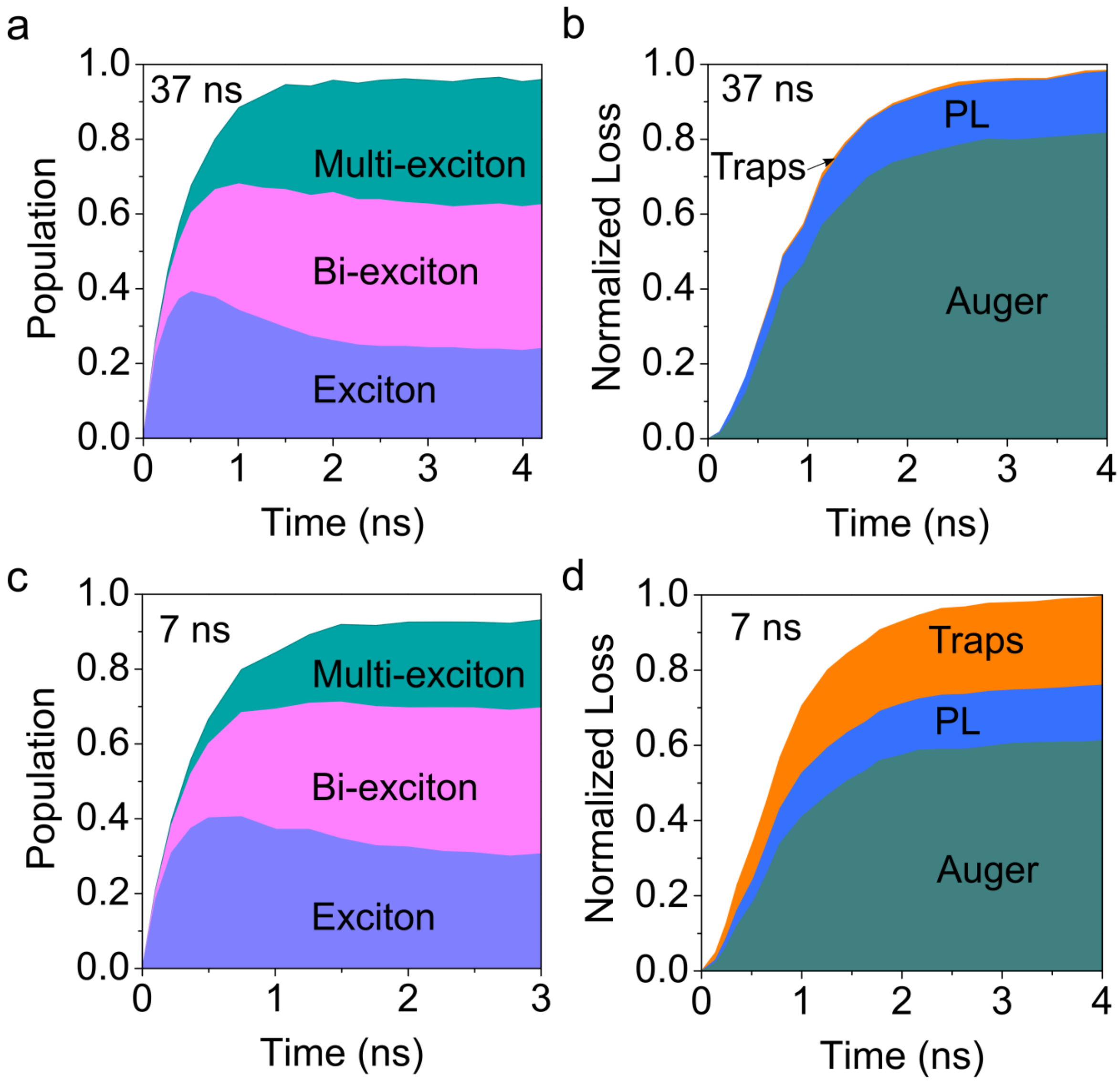

Supplementary Figure 4: Monte Carlo simulations for a PL lifetime of 37 ns and 7 ns. The contribution of single exciton, biexcition and multi-exciton population (a,c) and the contribution of recombination losses from Auger, photoluminescence (PL), and traps (b,d). The PL lifetime of 37 ns represents organic ligand capped dots (a,b) whereas 7 ns represents inorganic ligand capped dots.

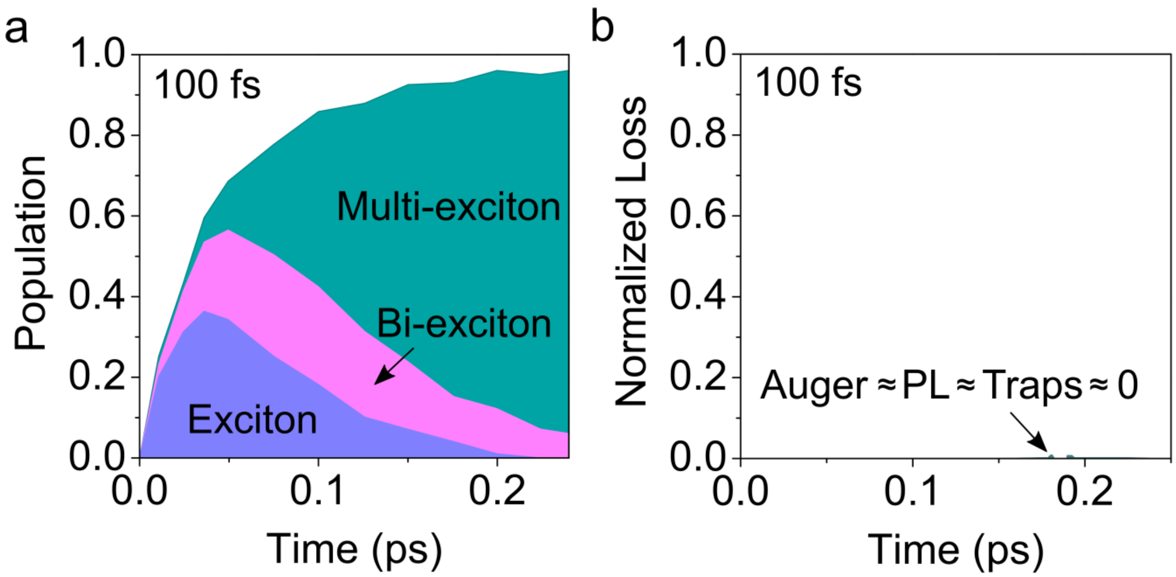

Supplementary Figure 5: Monte Carlo simulations for a 100 fs pulse. a) Population of multi-excitons, bi-excitons, and single excitons, and b) normalized loss from Auger, photoluminescence (PL) and trap-assisted recombination.

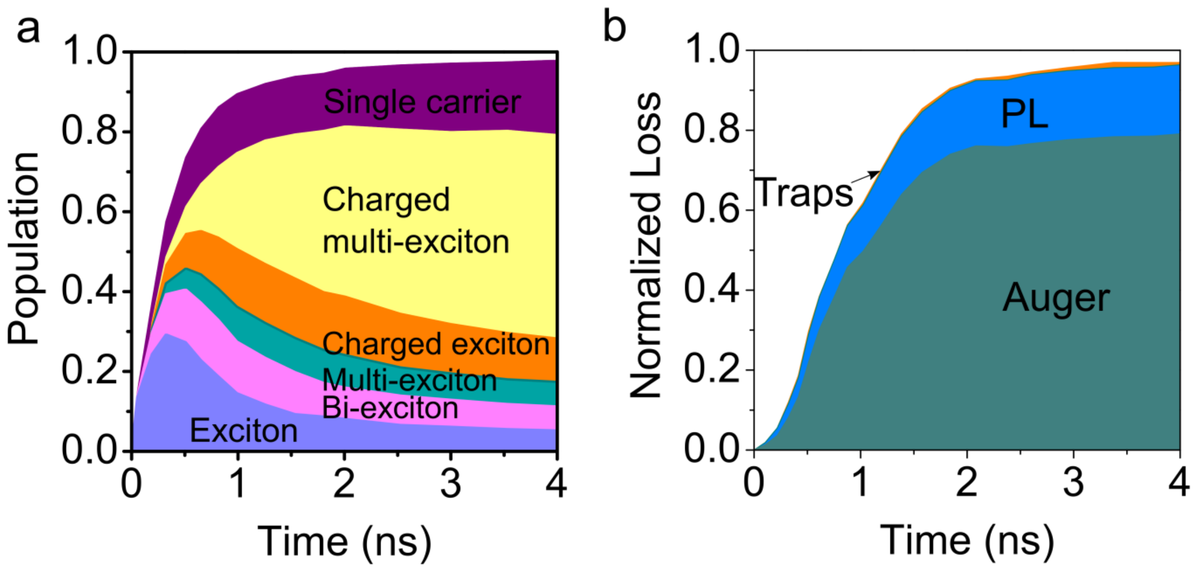

Supplementary Figure 6: Monte Carlo simulations taking into consideration dissociation. The contributions of single exciton, biexcition, multi-exciton, charged exciton, charged multi-exciton, and single carrier (hole or electron) population (a) and the contribution of recombination losses from Auger, photoluminescence (PL) and traps (b). The PL lifetime is 7 ns. The shorter PL lifetime in inorganic halide capped CQDs is an indication of exciton dissociation. Monte Carlo simulation taking into consideration dissociation shows the single-exciton, biexciton, multi-exciton, charged exciton, charged multi-exciton, and single carrier (hole or electron) populations as a function of time (Supplementary Figure 6a). The PL lifetime is 7 ns. The recombination loss is due to Auger recombination and photoluminescence (Supplementary Figure 6b). The loss due to traps is nearly zero.

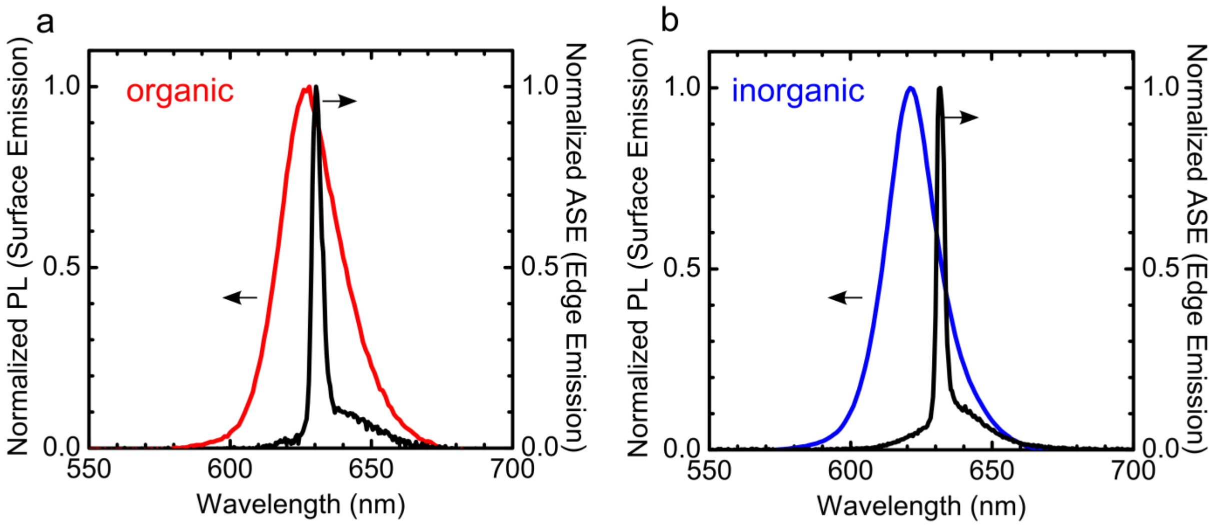

Supplementary Figure 7: Emission collected from the surface and edge of the film. The ASE emission data shown in Fig. 2a and Fig 2b was collected at the edge of the sample. The PL spectra collected from the surface of the sample (Supplementary Figure 7) shows that the ASE position is red-shifted from the PL peak position for both the organic ligand-capped and inorganic halide- capped CQD films, indicating a type I structure. [1]

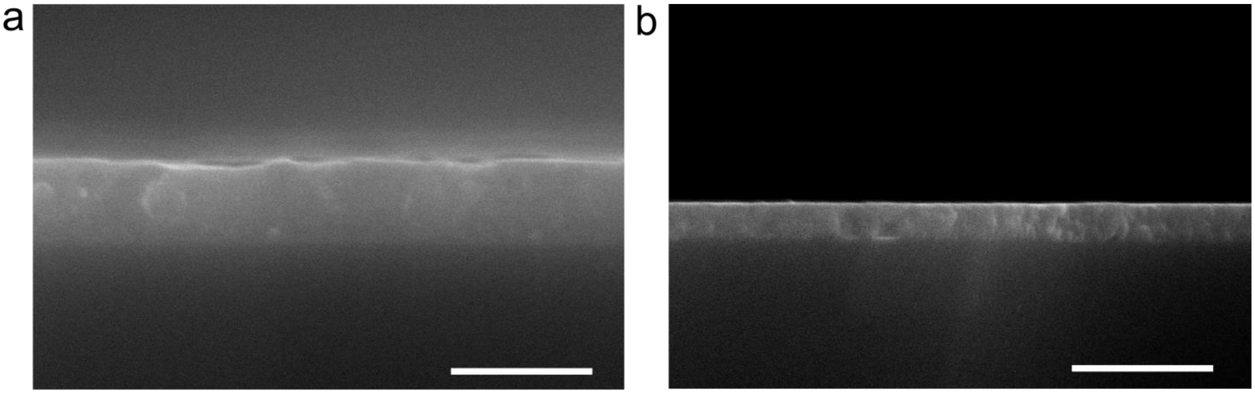

Supplementary Figure 8: Cross section SEM images of the films used ASE threshold measurements. (**a**) SEM cross section of the organic-ligand capped film. (**b**) SEM cross section of the inorganic-halide capped CQD films. The thickness is 298 nm ±15nm for the organic-ligand capped and 119nm ± 5 nm for the inorganic-halide capped films corresponding to nearly equal absorption at the pump wavelength of λ~355 nm. Scale bars, 500 nm.

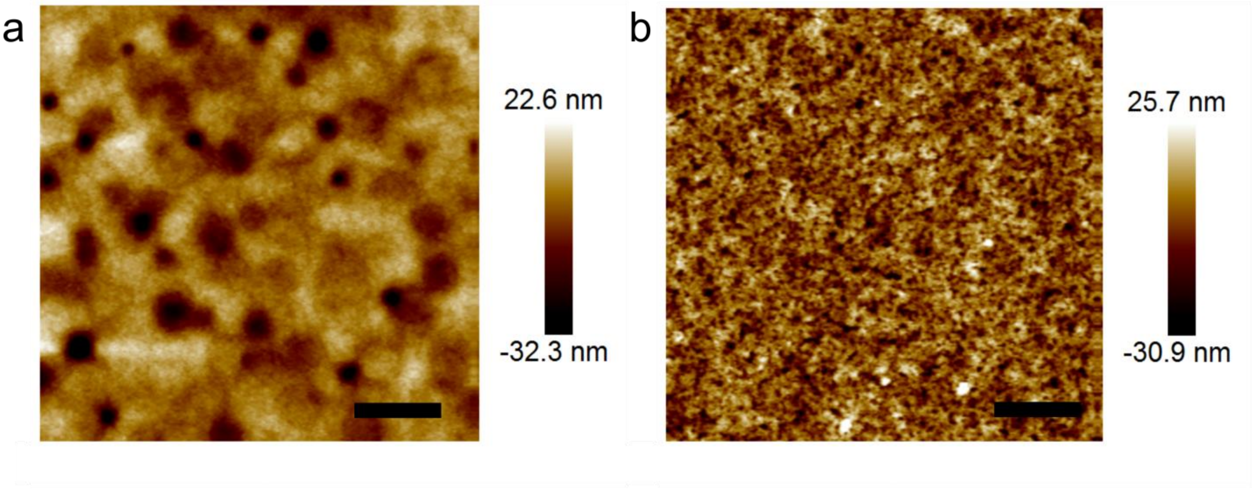

Supplementary Figure 9: Surface morphology. a, AFM profile of the organic-ligand capped CQD film. **b**, AFM profiles of the inorganic-halide capped CQD film. The scale bars are 1 μm. The organic-ligand capped film has a roughness of R_q=8.22 nm whereas the inorganic-halide capped film has a roughness of R_q=6.36 nm.

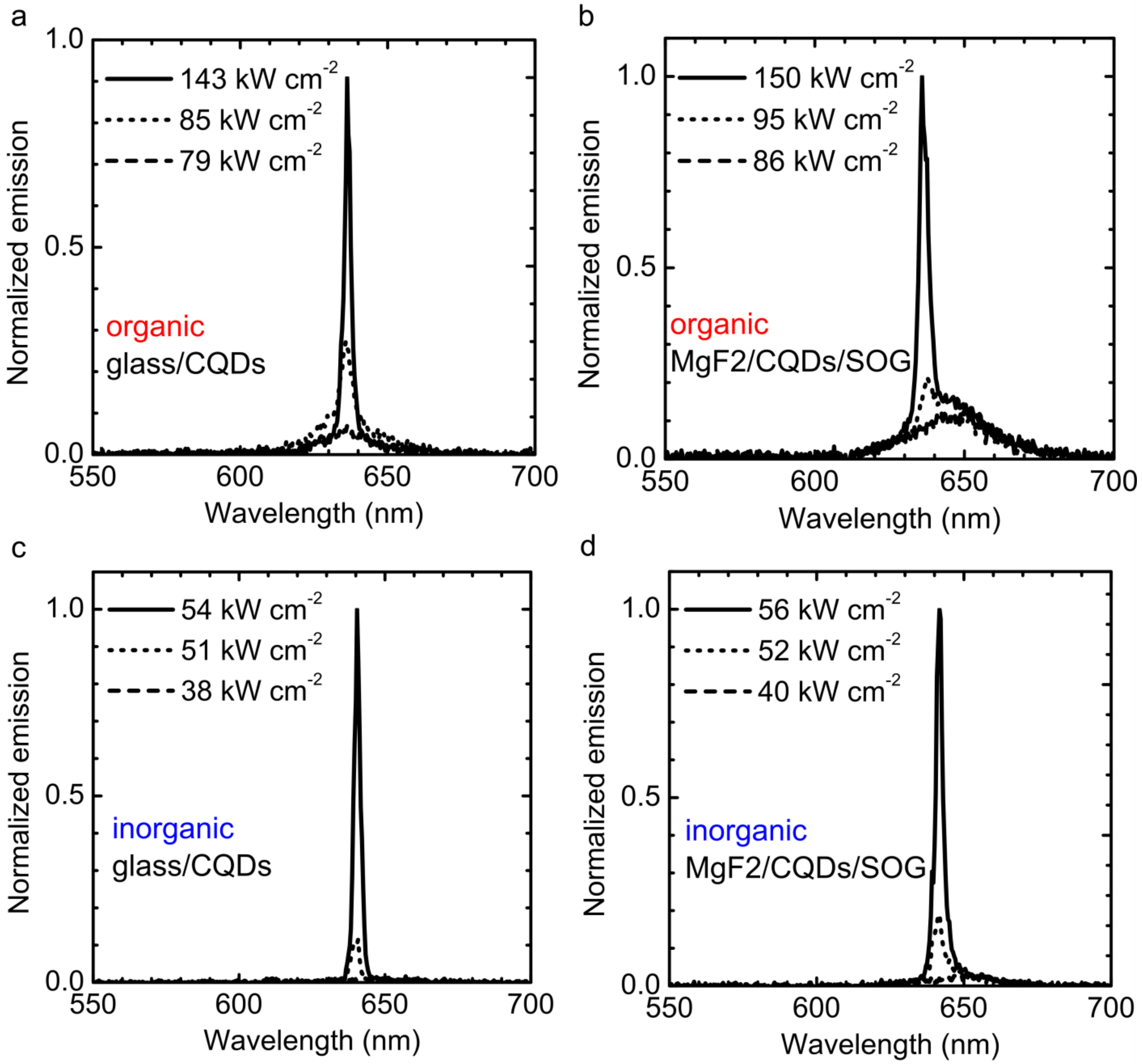

Supplementary Figure 10: Substrate-dependent ASE threshold. The ASE threshold for an organic-capped CQD film on glass substrate (a) is about 12% lower than that of the MgF_2 substrate/CQD/spin-on-glass (SOG) structure (b) used in the DFB. The ASE threshold for inorganic-capped CQD film is approximately the same on glass substrate as that of the MgF2/CQD/spin-on-glass structure. Therefore, the ASE threshold of the inorganic CQD film is preserved in the MgF2/CQD/spin-on-glass structure.

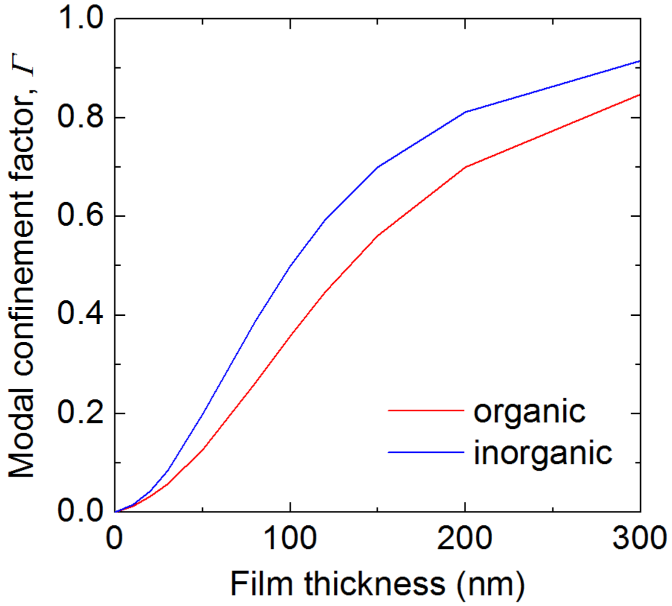

Supplementary Figure 11: Modal confinement factor simulation for the MgF_2/CQD/spin-on-glass structure as a function of CQD film thickness. The modal confinement factor simulation for the MgF_2/CQD/spin-on-glass structure (Supplementary Figure 11) again shows higher confinement factor for the inorganic CQD than the organic CQD films. Note that the spin-on-glass did not help to lower ASE threshold (Supplementary Figure 10) but was used to as a protective layer from condensation during characterization.

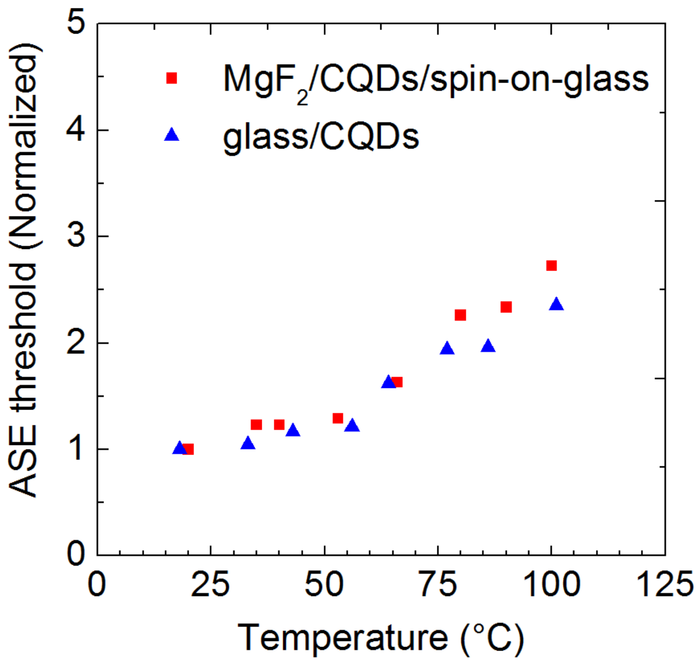

Supplementary Figure 12: Temperature dependent ASE threshold. The ASE threshold for inorganic capped CQDs on glass substrate and MgF2 substrate coated with spin-on-glass. The ASE threshold follow the same trend on both substrates.

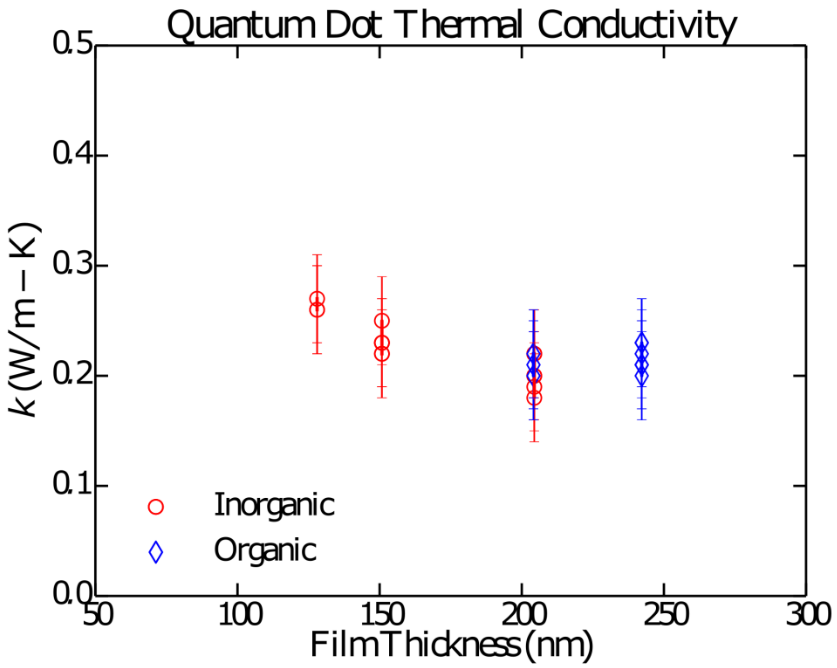

Supplementary Figure 13: Individual thermal conductivity measurements across samples of different thicknesses for organic-ligand and inorganic-halide capped quantum dot films. The error bars denote standard deviation.

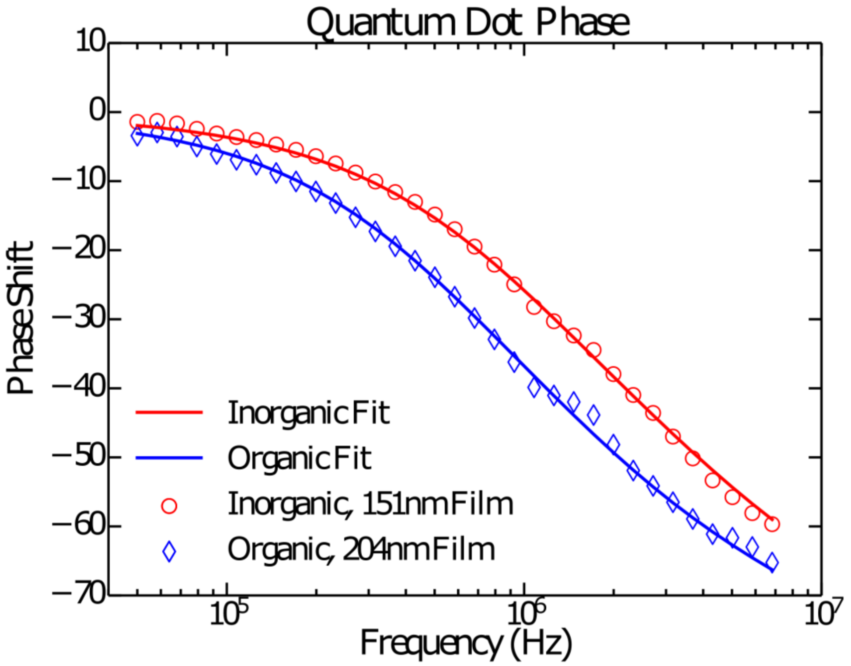

Supplementary Figure 14: The pump-probe phase shift versus heating frequency experimental data (discrete points) and analytical fit (lines) for organic-ligand and inorganic-halide capped quantum dot films.

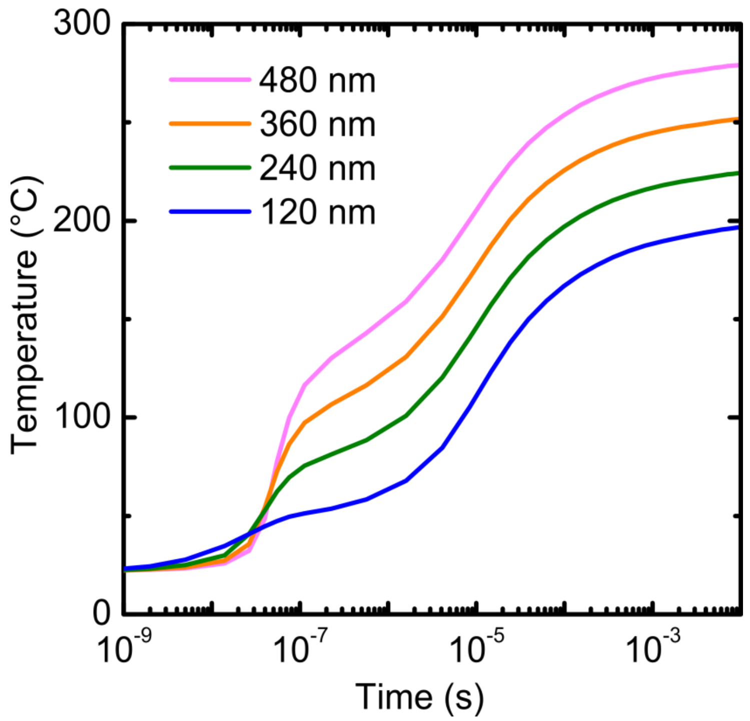

Supplementary Figure 15: Simulated temperature as a function of time for four different film thicknesses with equal OD.

Supplementary Tables

Supplementary Table 1| Summary of input parameters for thermal conductivity calculations.

	L_{Au}	k_{Au}^{*}	c_{Au}	ρ_{Au}	L_{CQD}	k_{CQD}	c_{CQD}	ρ_{CQD}
Inorganic	94 ± 5% nm	147 ± 5% W $m^{-1}K^{-1}$	130 J $kg^{-1}K^{-1}$	19300 kg m^{-3}	128-204 ± 5% nm	Fit	2633 ± 25% J $kg^{-1}K^{-1}$	374 ± 25% kg m^{-3}
Organic	94 ± 5% nm	69 ± 5% W $m^{-1}K^{-1}$	130 J $kg^{-1}K^{-1}$	19300 kg m^{-3}	204-242 ± 5% nm	Fit	1790 ± 25% J $kg^{-1}K^{-1}$	591 ± 25% kg m^{-3}

* Measured by Wiedemann-Franz

Supplementary Table 2| Input parameters for each material used in thermal simulations.

Substrate	Thermal conductivity (*k*), W $m^{-1}K^{-1}$	Density (ρ), kg m^{-3}	Heat capacity (*c*), J $kg^{-1}K^{-1}$
Inorganic CQDs	0.24	2633	374
Organic CQDs	0.22	1790	591
Glass	1.4	2500	750
MgF2	30 (cross-plan)<br>21 (in-plane)	3180	1003
Sapphire	42	3970	951
Si	148	2330	750
Al	237	2689	951

SUPPLEMENTARY NOTE 1

Monter Carlo Simulations

Monte Carlo simulations under constant-temperature conditions (Supplementary Figure 4) show the transient behavior of single, biexciton, and multi-exciton populations above gain threshold (excitation power density of 50 kW cm^{-2}) under the influence of an Auger lifetime of 700 ps. The single excition, biexciton, and multi-exciton populations are observed to reach steady-state within approximately 2 nanoseconds for both PL lifetimes of 37 ns (a) and 7 ns (c). For a PL lifetime of 37 ns, the normalized loss can be attributed to Auger recombination and photoluminescence (PL) radiative recombination (Supplementary Figure 4b). Loss due to trap-assisted recombination is nearly zero. However, for a PL lifetime of 7 ns (Supplementary Figure 4d), trap-assisted recombination is greater than PL recombination. The total heat generated from Auger and trap-assisted recombination is approximately the same for PL lifetimes of the 37 ns and 7 ns. Monte Carlo simulations were also performed for a 100 fs pump pulse (Supplementary Figure 5). The single-exciton, biexciton, and multi-exciton populations reach steady-state within approximately 0.25 ps after excitation (Supplementary Figure 5a). At this time scale, the Auger, PL, and trap-assisted recombination losses are nearly zero (Supplementary Figure 5b).

Thermal analysis

Our thermal simulations are performed on a continuum level, therefore the thermal excitation is treated as a heat generation term. This heat generation term was determined using experimentally obtained parameters such that: Heat Generation = ASE Threshold *Absorption * [PLQY*(2.80eV - 1.94eV)/2.80eV + (1-PLQY)], where ASE threshold is in average peak power density, and PLQY is the photoluminescence quantum yield at ASE threshold pump density (Auger recombination reduces the PLQY to about 20-30% for organic films and 10-20% for inorganic films at ASE threshold). This bracketed term accounts for radiative (blue to red down conversion) and non-radiative heat generation.

Heat generation was activated at the beginning of the thermal simulation (t = 0) and held constant for the duration of the simulation and simulates a single, isolated pulse.

After instantaneous illumination of a quantum dot film top-surface using pump light, unconverted energy generates heat that will diffuse throughout the film and reach the substrate after τ ~ 70 ns. In other words, the ability for the substrate to effectively remove thermal energy becomes important for applications where lasing lasts longer than ~70 ns. This onset is evident in Fig. 4c where films on substrates with different thermal conductivity begin to diverge after a few thermal time constants.

Here the thermal time constant is $\tau = L^2\rho c/k = 72$ ns, where the quantum dot film thickness is L~120 nm, thermal conductivity is k = 0.2 $Wm^{-1}K^{-1}$, and the term density*specific heat is ρ*c = 1e6 $Jm^{-3}K^{-1}$.

The photoluminescence quantum yield was determined using a reported method.[2] An integrating sphere and calibrated Ocean optic USB 2000 spectrometer were used to collect spectra. A low-power continuous wave 442 nm laser was used as low-power pump source and a pulsed (355 nm, 100 HZ, 1 ns pulse width) laser was used for high peak power pump excitation. The pump intensity was controlled using a rotating neutral density filter.

SUPPLEMENTARY METHODS

Refractive index measurement

The refractive index of the CQDs was measured by two methods: ellipsometry and by minimum thickness required to achieve amplified spontaneous emission. CQDs show minimal interference patterns in the wavelength range of λ=300 nm to 3300 nm so refractive index could not be determined by transmission/reflectance maximum and minimum.

Method 1: Ellipsometry

Ellipsometry data was obtained using a spectroscopic ellipsometer (SOPRA GES-5E) at an incident angle of 56°. Samples consist of a CQD film on glass substrate where the back-side of the glass was roughened to avoid back-surface reflections. To fit the ellipsometric data, a simple stack of void/film/substrate was used where the film was modeled using a Cauchy dielectric function dispersion law. 3 Lorentz peaks were added which corresponded to the absorption peaks obtained by UV-VIS spectroscopy and the fit was taken over a photon energy range of

1.4eV to 2.5 eV. The film thickness was determined from cross-sectional SEM and fixed during model fitting. The resulting refractive at λ=630 nm was 1.97 ± 0.05 (standard error) for the inorganic-halide capped film and 1.74 ± 0.05 for the organic-ligand capped film.

Method 2: Minimum thickness required to achieve ASE

When ASE is measured for a CQD film on glass substrate, stimulated emission is generated by light propagating laterally within the film. Therefore, no ASE is observed for film that is too thin to sustain a mode. For organic-ligand capped dots a minimum film thickness of 120 nm ± 10nm is required to achieve ASE (threshold: 188 μJ cm^{-2}), whereas in inorganic-halide capped dots a minimum thickness of 72nm ± 5 nm is required to achieve ASE (threshold:98 μJ cm^{-2}) (Supplementary Figure 1a). Using a mode solver (Lumerical Mode Solutions) we can derive the refractive index as the minimum index required to achieve modal gain > 0 (Supplementary Figure 1b). The material gain is 423 cm^{-1} at the ASE threshold of 62 μJ cm^{-2} for the organic-ligand capped dots, and 486 cm^{-1} at the ASE threshold is 39μJ cm^{-2} (Fig.1c) for the inorganic-ligand capped dots. The refractive index is calculated to be ~1.74 for the organic-ligand capped CQDs and ~1.92 for the inorganic-ligand capped CQDs. These values agree within error with the refractive index determined by ellipsometer.

Thermal conductivity measurement

The Frequency Domain Thermoreflectance (FDTR) technique is sensitive to the thin quantum dot layers since they have a higher thermal resistance than the other layers (i.e. gold, silicon), and hence dominate the temperature response at the surface. The densities and specific heat capacities of the quantum dot layers, however, are not well characterized inputs that are

necessary for the analytical model. We calculated the volume-weighted density and the mass-weighted specific heat capacity of both the organic-ligand and inorganic-halide capped dots using the bulk constituent properties to approximate these values. Specifically, the CQD density was calculated by

$$\rho_i = \sum_j V_j \rho_j / \sum_j V_j, \qquad (1)$$

where ρ_i is the CQD density of either the organic-ligand capped or inorganic-halide capped dots, V_j is the volume of component j in the CQD and ρ_j is the bulk density of component j. Similarly, the CQD specific heat capacity was calculated by

$$c_i = \sum_j V_j \rho_j c_j / \sum_j V_j \rho_j, \qquad (2)$$

where c_j is the bulk specific heat capacity of component j. The uncertainty in these parameters dominate the uncertainty of our thermal conductivity measurements in comparison to other parameters (Au film thermal conductivity, Au film thickness, CQD film thickness, laser spot diameter).

Although our measurements agree well with previous measurements of similar colloidal nanoparticles with organic/inorganic ligands with thermal conductivities between 0.1-0.3 W $m^{-1}K^{-1}$, there was no measured increase in thermal conductivity of the inorganic ligand nanoparticles versus the organic ligand nanoparticles as shown previously[3,4]. We believe that this is most likely due to an incomplete removal of the Oleic acid during the halide exchange for the inorganic-halide CQD. We speculate that this residual Oleic acid provides enough interfacial resistance between quantum dots to reduce the thermal conductivity of the film.

Below are the parameters and associated uncertainties used for the analytical model of the system for which the thermal conductivity of the quantum dot films was measured. The thermal conductivity values for samples of different thicknesses are plotted below in Supplementary Figure 13 and the phase shift versus heating frequency data for two representative fits are plotted in Supplementary Figure 14. The uncertainty in thermal conductivity for each parameter was calculated by fitting the thermal conductivity with one of the input parameters perturbed by its uncertainty to produce a deviation in thermal conductivity. Then the square root of the sum of the square deviations in thermal conductivity for each parameter yields an overall uncertainty in thermal conductivity. [5]

Monte Carlo simulations

Monte Carlo simulations were performed on a 20x20x20 grid of QDs with excitation intensities adjusted to above-transparency threshold in the ensemble of quantum dots, i.e. the dots with 1 exciton are assumed transparent and the number of bi-exciton or multiple exciton-populated dots should exceed the amount of the remaining empty dots. The same power per pulse was used for all simulated cases. The time step was chosen so that the number of adsorbed photons per timestep is less than 1 (for the transparency threshold this corresponds to 36 fs). Other processes such as Auger recombination and radiative recombination are significantly slower than this time step. The PL lifetime of 37 ns (Supplementary Figure 3) and Auger lifetime of 700 ps were used following the experimental values. Trap recombination was also taken into account, with lifetimes derived from PLQY values. For the Cl-exchanged films with the 7 ns lifetime two cases were considered: a) the lifetime speedup is due to increased trapping losses, and b) the shorter lifetime is due to fast exciton dissociation. The Auger recombination rate of multiexcitons was

increased proportionally to their multiplicity.[6] Simulations show that Auger recombination accounts for most of the losses within the film and the remaining are due to recombination from PL or traps (Supplementary Figure 4b and 4d).

SUPPLEMENTARY REFERENCES

1. Klimov VI, *et al.* Single-exciton optical gain in semiconductor nanocrystals. *Nature* **447**, 441-446 (2007).

2. de Mello JC, Wittmann HF, Friend RH. An improved experimental determination of external photoluminescence quantum efficiency. Advanced Materials 9, 230-232 (1997).

3. Ong W-L, Rupich SM, Talapin DV, McGaughey AJH, Malen JA. Surface chemistry mediates thermal transport in three-dimensional nanocrystal arrays. *Nat Mater* **12**, 410-415 (2013).

4. Ong W-L, Majumdar S, Malen JA, McGaughey AJH. Coupling of Organic and Inorganic Vibrational States and Their Thermal Transport in Nanocrystal Arrays. *The Journal of Physical Chemistry C* **118**, 7288-7295 (2014).

5. Malen JA, Baheti K, Tong T, Zhao Y, Hudgings JA, Majumdar A. Optical Measurement of Thermal Conductivity Using Fiber Aligned Frequency Domain Thermoreflectance. *Journal of Heat Transfer* **133**, 081601-081601 (2011).

6. Klimov VI, McGuire JA, Schaller RD, Rupasov VI. Scaling of multiexciton lifetimes in semiconductor nanocrystals. *Physical Review B* **77**, 195324 (2008).
